# The Injury-Related Activation of Hedgehog Signaling Pathway Modulates the Repair-Associated Inflammation in Liver Fibrosis

**DOI:** 10.3389/fimmu.2017.01450

**Published:** 2017-11-06

**Authors:** Xin Shen, Yu Peng, Hanmin Li

**Affiliations:** ^1^Department of Laboratory Medicine, Hubei University of Chinese Medicine, Wuhan, China; ^2^Department of Information Engineering, Hubei University of Chinese Medicine, Wuhan, China; ^3^Hepatic Disease Institute, Hubei Provincial Hospital of Traditional Chinese Medicine, Affiliated Hospital of Hubei University of Chinese Medicine, Hubei Province Academy of Traditional Chinese Medicine, Wuhan, China

**Keywords:** hedgehog signaling pathway, repair-associated inflammation, immune-modulatory action, hedgehog pathway inhibitor, liver fibrosis

## Abstract

Liver fibrosis is a wound healing response initiated by inflammation responding for different iterative parenchymal damages caused by diverse etiologies. Immune cells, which exert their ability of either inducing injury or promoting repair, have been regarded as crucial participants in the fibrogenic response. A characteristic feature of the fibrotic microenvironment associated with chronic liver injury is aberrant activation of hedgehog (Hh) signaling pathway. Growing evidence from a number of different studies *in vivo* and *in vitro* has indicated that immune-mediated events involved in liver fibrogenesis are regulated by Hh signaling pathway. In this review, we emphasize the impacts of injury-activated Hh signaling on liver fibrogenesis through modulating repair-related inflammation and focus on the regulatory action of aberrant Hh signaling on repair-related inflammatory responses mediated by hepatic classical and non-classical immune cell populations in the progression of liver fibrosis. Moreover, we also assess the potentiality of Hh pathway inhibitors as good candidates for anti-fibrotic therapeutic agents because of their immune regulation actions for fibrogenic liver repair. The identification of immune-modulatory mechanisms of Hh signaling pathway underlying the fibrotic process of chronic liver diseases might provide a basis for Hh-centered therapeutic strategies for liver fibrosis.

## Introduction

The liver possesses profound regenerative potential. Following acute injury, even if a large part of this organ is destroyed, the liver is capable of restoring its complete mass and original architecture in a comparatively short time. In contrast, chronic progressive injury to the liver will induce repetitive tissue damage and result in impaired regenerative capacity marked by sustaining inflammation and extracellular matrix remodeling, eventually culminating in fibrosis and cirrhosis (1). Regardless of the underlying cause, liver fibrosis is viewed as a wound healing response initiated by inflammation responding for different iterative parenchymal damages (2). This injury-induced inflammation has been proved as a double-edged sword for liver regeneration and repair: moderate and resolved inflammatory responses are beneficial to the liver as they exert consistent hepato-protective effects, contribute to tissue repair, and promote the recovery of tissue homeostasis, whereas excessive and persistent inflammatory responses not only aggravate liver injury as triggered by a wide array of hepatotoxic insults but also promote fibrosis and even hepatic carcinogenesis (3). Therefore, the injury-induced inflammation has an important impact on regulating liver regeneration and fibrosis. The mechanisms that govern fibrogenic liver repair are complex and interactions between various types of innate and adaptive immune cells with stromal cells mediated by cytokines, growth factors, or modified matricellular proteins are implicated (4). Understanding the immunological mechanisms of liver fibrosis provides the foundation for designing new therapies that do not depend on the underlying cause.

Several signal transduction pathways, which direct cell fate decisions including cell proliferation, differentiation, and migration in the phase of embryogenesis, are reactivated in the case of liver injury and are verified to regulate liver regeneration and repair. One such signal transduction pathway is hedgehog (Hh) signaling pathway. Hh pathway, initially verified in the *Drosophila* fruit fly, is a highly conserved signaling pathway, which is considered to play an important regulatory role in embryonic development and adult tissue remodeling of multiple tissues and organs, including the liver (5). In particular, evidence from studies in adult rodents and humans indicates that the injury-related activation of Hh signaling coordinates several fundamental facets of fibrogenic liver repair, such as the amplification of liver progenitors, the aggregation of hepatic myofibroblasts, repair-related inflammatory responses, and vessel remodeling (6). Noticeably, the regulation of repair-related inflammatory responses in chronic liver injury by Hh signaling pathway extends current understanding of this signaling pathway’s immune-modulatory actions, since the immune regulation of Hh signaling pathway initially reflects that Hh ligands have been identified as key modulators of many phases of T-cell development in the thymus (7). Growing evidence from a number of different studies *in vivo* and *in vitro* has indicated that immune-mediated events involved in liver injury and fibrogenesis are regulated by Hh signaling pathway. Hence, in this review, we emphasize that Hh, as a non-immune factor existing on the microenvironment of tissue repair and regeneration, has been shown to modulate immunological aspects in the fibrotic process of chronic liver diseases. More importantly, these data contribute to characterize how microenvironmental cues influence immune cells to modulate liver tissue remodeling. Furthermore, we also discuss that Hh pathway inhibitors are good candidates for anti-fibrotic therapeutic agents because of their immune-regulation actions for fibrogenic liver repair.

## Overviews of Hh Signaling Pathway in Liver Fibrosis

Hedgehog signaling pathway is a highly complex signaling pathway and mainly involves canonical signaling pathway and non-canonical signaling pathway. There are three types of Hh ligands, Sonic Hedgehog (Shh), Indian Hedgehog (Ihh), or Desert Hedgehog (Dhh) in Hh signaling pathway (8). In canonical signaling pathway, the binding of Hh ligands (Shh, or Ihh, or Dhh) to the transmembrane receptor patched (Ptc) triggers Ptc to relieve its tonic inhibition of Smoothened (Smo) (9). Released Smo then aggregates in the primary cilium, which contributes to the nuclear localization of Gli transcription factors (9). There are three glioma-associated oncogene homolog (Gli) transcription factors (Gli1, Gli2, and Gli3), all of which are zinc finger proteins comprising a DNA-binding domain and five tandem C_2_H_2_ zinc finger motifs (8). Meanwhile, there is an activator domain (GliA) at the C-terminus of all three Gli transcription factors and is a repressor domain (GliR) at the N-terminus of Gli2 and Gli3 (8). In mammals, the function of three Gli transcription factors is partly overlapping but also contrary. Gli2 is characterized as the main activator of Hh signaling, whereas Gli3 is accountable for the repression function in response to Hh signaling (9). In the nucleus, Gli2 or Gli3 binds to DNA and then modulates the transcription of numerous Hh target genes. As for Gli1, it seems to be a signaling amplifier of the Gli2-mediated transcriptional response (9). Suppressor of fused (SUFU) is an important negative modulator of Hh signaling. Lack of Hh ligands, the binding of SUFU to Gli transcription factors facilitates the ubiquitination and degradation of Gli transcription factors, and correspondingly inhibits the activation of Hh signaling pathway. In addition, there is a vertebrate-specific feedback antagonist of Hh signaling, Hh-interacting protein (Hhip), which competes with Ptc for binding to Hh ligands and then attenuates Hh signaling through a negative regulatory feedback loop (10). Besides the aforementioned canonical signaling pathway, numerous studies suggest that there are non-canonical signaling pathways. For example, Ptc is shown to modulate cell cycle through cyclin B1, which do not require Smo and Gli transcription factors (11, 12). Moreover, the downstream effect of Smo also is demonstrated to be mediated by the activation of small GTPases independent of Gli transcription factors (13).

During fibrogenic liver repair caused by various types of chronic injury, emerging evidence has demonstrated a critical role of canonical Hh signaling, which is supported that conditional deletion of Smo in α-SMA^+^ myofibroblasts inhibited liver fibrosis (14). Moreover, multiple injury-associated factors participating in liver fibrogenesis have been shown to directly activate Gli transcription factors, which include platelet-derived growth factor (PDGF) (15), transforming growth factor β1 (TGF-β1) (16), epidermal growth factor (EGF) (17), and insulin-like growth factors (18). Conversely, Hh transcription factors have been demonstrated to regulate the activities of target genes in other signaling pathways. For example, Gli could regulate transcription of a TGF-β/Smad signaling pathway target gene Snail (19) and also impact the expression of Wnt signaling pathway modulators, such as Wnt5a (an activator of Wnt signaling pathway) and soluble frizzled receptor-1 (sFRP1, an inhibitor of Wnt signaling pathway) (20). Therefore, Hh signaling pathway, as one of key regeneration signaling (14) and fibrogenic signaling pathways (21), is part of a complex signaling network that involves other essential signaling pathways controlling cell fate acquisition, such as TGF-β/Smad and Wnt signaling pathways. Within this signaling network, Hh signaling interacts with these signaling pathways, which orchestrates global changes in the phenotypes and function of Hh-responsive cells and regulates the injured liver regeneration and fibrogenesis (22).

## Roles of Injury-Related Activation of Hh Signaling Pathway in Liver Fibrosis

In healthy adult liver, there is lack of detectable activity of Hh pathway because of low Hh ligand expression and relatively high Hhip expression (22). During liver injury, injury stimulates liver epithelial cells, including mature hepatocytes (23, 24) and cholangiocytes (25), to produce and release Hh ligands, and simultaneously represses sinusoidal lining cell expression of Hhip (17, 26, 27). Then, Hh ligands activate the Hh signaling pathway in surrounding Hh-responsive cells, including quiescent hepatic stellate cells (Q-HSCs), liver progenitors, liver sinusoidal endothelial cells (LSECs), and immune cells, through ligand–receptor interactions. Meanwhile, most of these Hh-responsive cells are also Hh-producing cells, which are capable of producing Hh ligands in response to injury-induced Hh ligands or other injury-associated factors [e.g., PDGF-BB (15), TGF-β1 (16), and EGF (17)] in turn. This triggers a cascade causing excessive activation of Hh signaling pathway. Because of the extensive enrichment of Hh ligands, the proximity of Hh-producing and Hh-responsive cells in anatomical structure, with the addition of the complex autocrine and paracrine Hh signaling loops in the remodeling and reconstruction process of injured liver, Anna Mae Diehl’s group (28) and Grzelak and colleagues (29) put forward the concept of the intrahepatic signaling niches of Hh. In this injury-related intrahepatic signaling niches of Hh, Hh signaling as a mediator has an impact on the interactions between Hh-producing cells and Hh-responsive cells, as well as their biological functions in liver regeneration and repair (30).

Existing data suggest that determining whether Hh signaling induces adaptive responses or maladaptive responses to liver injury depends on the equilibrium between its regulatory effect on the expansion of progenitor cells and its impact on promoting liver inflammation and fibrosis (22). Hh signaling activated by injury is critical for the regeneration of liver tissue, which is verified that treatment with Smo antagonist interfered with the accumulation of liver progenitors, suppressed the proliferation of hepatocytes and cholangiocytes, blocked the regeneration of liver tissues, and dramatically reduced the survival of normal mice after 70% partial hepatectomy (31). However, due to persistent liver injury, the sustained expression of Hh signaling perpetuates the expansion of cell types, which are accountable for the fibrotic progression of chronic liver diseases, including myofibroblasts, activated sinusoidal endothelial cells, immature liver epithelial cells, and certain types of immune cells (15, 16). Especially, myofibroblasts are viewed as the major source of fibrillar collagen forming the fibrous scar in fibrotic liver tissues, and the myofibroblastic transformation of resident Q-HSCs is commonly considered the primary origin of myofibroblasts in most cases of chronic liver injury (32). Study showed that Hh ligands can activate hepatic stellate cells (HSCs) and induce their transdifferentiation from a quiescent phenotype into a myofibroblastic phenotype responsible for matrix deposition (22). Moreover, the activation of Hh pathway can inhibit apoptotic signals, enhance the viability and proliferative capacity of myofibroblastic HSCs (MF-HSCs), and stimulate additional production of endogenous Hh ligands in an autocrine or paracrine manner, which drives a positive feedback loop to amplify Hh signaling (17). Meanwhile, accumulating Hh ligands also induce the generation of hepatic myofibroblasts by non-conventional means. This mainly reflects on the following two aspects: first, activating Hh signaling can induce the secretion of monocyte chemoattractant protein 1 (MCP-1) and IL-13, which can chemoattract circulating monocytes/fibrocytes derived from bone marrow into injury liver and promote the transformation of monocytes into fibrocytes, respectively (33, 34). Second, growing evidence shows that Hh signaling can induce epithelial–mesenchymal transition (EMT) in certain liver epithelial cells, such as immature ductular cells (35), as part of repair following chronic injury in adult liver. Therefore, excessive Hh signaling plays a major role in hepatic accumulation of myofibroblasts in liver fibrogenesis, and pharmacologic inhibition of Hh signaling has been shown to alleviate liver fibrosis by abolishing the accumulation of myofibroblasts and resolving collagen matrix in the model of chronic cholestatic liver injury (36).

## Modulating the Repair-Associated Inflammation in Liver Fibrosis by Hh Signaling Pathway

As mentioned above, liver fibrosis is commonly preceded by chronic inflammation (37). Excessive Hh signaling activated by chronic injury in the progression of liver fibrosis has been shown to modulate hepatic accumulation of inflammatory cells. Hh ligands are produced by a variety of cells including dying hepatocytes, cholangiocytes, (myo-)fibroblasts, and immune cells, and then stimulate immature cholangiocytes to secrete chemokines through autocrine or paracrine modes of action, which can further recruit, attract and retain immune cells (e.g., macrophages, monocytes, neutrophils, and lymphocytes) into the injured liver tissues (33). This might explain why fibrogenesis takes place under the circumstance of chronic inflammation, and this inflammation throughout the whole process of fibrogenic liver repair is consequently described as the “repair (fibrosis)-associated inflammatory response” (6, 37).

**Figure 1:**
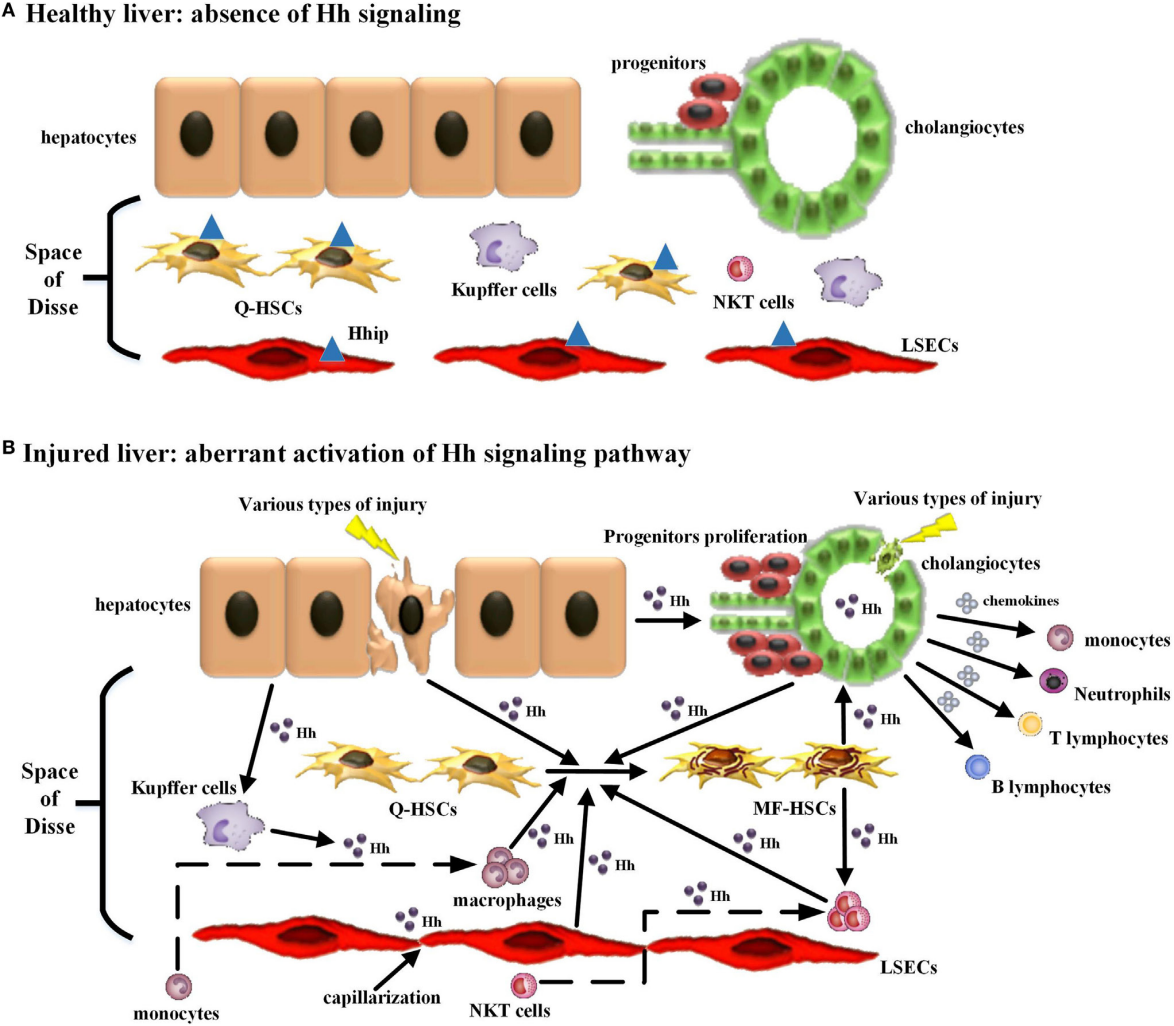
Modulating the repair-associated inflammatory response in liver fibrosis by the injury-related activation of hedgehog (Hh) signaling pathway. **(A)** In healthy liver, there is lack of detectable activity of Hh pathway because of low Hh ligand expression and relatively high Hh ligand antagonist (Hhip) expression in liver sinusoidal lining cells [quiescent hepatic stellate cells (Q-HSCs) and liver sinusoidal endothelial cells (LSECs)]. **(B)** In injured liver, various types of injury stimulate liver epithelial cells (mature hepatocytes and cholangiocytes) to produce and release Hh ligands, repress the expression of Hhip in liver sinusoidal lining cells, and then unleash a cascade that results in aberrant activation of Hh signaling pathway. The cell populations that are involved in liver inflammation, regeneration, and fibrogenesis are consequently expanded, and most of these cells are Hh-producing cells and/or Hh-responsive cells. The extensive enrichment of Hh ligands, the proximity of Hh-producing and Hh-responsive cells in anatomical structure, with the addition of the complex autocrine and paracrine Hh signaling loops consist of the injury-related intrahepatic signaling niches of Hh, which in turn has an impact on the roles of these cell populations, in particular immune cell population, during liver fibrogenesis. (1) Hh ligands can induce the transdifferentiation of Q-HSCs into myofibroblastic HSCs (MF-HSCs) and further stimulate the production of Hh ligands by MF-HSCs. (2) Hh ligands secreted by injured liver epithelial cells and MF-HSCs can promote the proliferation and survival of liver progenitor population, and then the accumulation of liver progenitor population contributes to the regeneration of the injured liver. (3) Hh ligands, as viability factors for natural killer T (NKT) cells, contribute to the recruitment of circulating NKT cells into the injured liver, prolong their intra-hepatic survival and increase their opportunities for the exposure to potential antigen-presenting cells. Moreover, pro-fibrogenic cytokines (e.g., IL-4 and IL-13) and OPN were secreted by Hh ligand-induced NKT cells, which, as well as Hh ligands produced by NKT cells themselves, further expand the population of liver myofibroblasts by promoting transdifferentiation of Q-HSCs into MF-HSCs. (4) Both Hh ligands and Hh-induced OPN can act as chemoattractants for monocytes and recruit bone marrow-derived monocytes into the injured liver. Furthermore, Hh ligands can autocrinally induce the alternative activation of macrophages while OPN can paracrinally stimulate the pro-inflammatory phenotype of hepatic macrophage, both of which contribute to the transdifferentiation of Q-HSCs into MF-HSCs. (5) Besides Hh ligands, Hh-induced cholangiocytes also secrete several chemokines that recruit different types of immune cells, such as neutrophils, monocytes/macrophages, and T and B lymphocytes, into the injured liver. These infiltrating inflammatory cells, in turn, produce more Hh ligands and amplify Hh signaling cascade. Meanwhile, these cells also produce pro-fibrogenic cytokines (e.g., IL-6, IL-4, IL-13, and transforming growth factor β1) that mediate duct injury and repair, and myofibroblastic differentiation of portal fibroblasts. (6) The accumulative effect of Hh ligands on LSECs is to promote LSEC capillarization. When capillarized, LSECs lose their abilities to maintain the quiescence of HSCs, and instead promote intrahepatic vasoconstriction and the development of fibrosis.

Moreover, immune cells participating in this repair-associated inflammation have been considered as pivotal players in the fibrogenic response and can exert their ability of either inducing injury or promoting repair (38). Most of these immune cell populations, consisting of classic immune cell populations [e.g., macrophages/Kupffer cells, natural killer T (NKT) cells, lymphocytes, neutrophils, and monocytes] and non-classical immune cell populations (i.e., non-hematopoietic cell populations, including LSECs, HSCs, hepatocytes, and cholangiocytes), are either Hh-producing cells or Hh-responsive cells (30) (Table 1). Increasing study further demonstrated that besides promoting the accumulation of inflammatory cells within the injured liver tissue, excessive Hh signaling in the fibrotic microenvironment also could modulate the phenotype of immune cells as well as their function. Hence, we will emphasize the impacts of injury-activated Hh signaling on liver fibrogenesis through modulating repair-related inflammation, and focus on the regulatory action of aberrant Hh signaling on repair-related inflammatory responses mediated by the aforementioned classical and non-classical immune cell populations in the progression of liver fibrosis (Figure 1).

**Table 1:** Profiles of hedgehog (Hh) signaling in major liver cells types.

Cell types	Hh-producing cells	Hh-responsive cells	Hh-targeted intervention responsive	Known Hh-dependent functions	Reference
**Classical immune cells**					
Natural killer T cells	P	R	Yes	Inducing the recruitment and hepatic migration; promoting the proliferation and survival; regulating the pro-fibrogenic effect	(33, 39–41)
Kupffer cells/Macrophages	P	R	Yes	Chemotaxis and the regulation of phenotypes	(42–44)
T lymphocytes	Not clear	R	Not clear	Being recruited into injured liver through chemokines released by cholangiocytes in a Hh-dependent manner	(33, 45–47)
B lymphocytes	Not clear	R	Not clear	Being recruited into injured liver through chemokines released by cholangiocytes in a Hh-dependent manner	(7, 33, 48, 49)
Neutrophils	Not clear	R	Not clear	Being recruited into injured liver through chemokines released by cholangiocytes in a Hh-dependent manner	(33, 50–52)
Myeloid-derived suppressor cells	Not clear	R	Not clear	Not clear	(53)

**Non-classical immune cells**					
Hepatocytes	Dying hepatocytes	R	Yes	Producing IGFI and IGFBP-1	(23, 54)
Cholangiocytes	P	R	Yes	Influencing viability and differentiation; inducing epithelial–mesenchymal transition (EMT) and the production of chemokines	(25, 33)
Liver sinusoidal endothelial cells	P	R	Yes	Regulating capillarization	(55)
Hepatic stellate cells (HSCs)	Activated HSCs	R	Yes	Inducing EMT	(17, 22)

### NKT Cells and Hh

Natural killer T cells are a functionally heterogeneous subset of non-conventional T lymphocytes displaying markers characterized of both NK cell and T cell, which can recognize non-classical MHC class I-like molecule CD1-presenting lipid antigens (56). The liver is a site of NKT cells enrichment, and the proportion of NKT cells in mouse liver lymphocytes is about 30–35%, whereas its proportion in rat and human liver lymphocytes is approximately 5–10%. These proportions of NKT cells in liver lymphocytes are significantly higher than that of NKT cells in peripheral blood lymphocytes (<5%) (57), suggesting that NKT cells have a fundamental impact on liver homeostasis and pathology. In the pathogenesis of liver chronic injury and fibrosis, the function of NKT cells appears to be complex. Activated NKT cells are able to play inhibitory effects on liver fibrosis *via* killing activated HSC directly (58) and producing anti-fibrotic mediators (IFN-γ and IL-30) indirectly (59, 60). Meanwhile, activated NKT cells also have pro-fibrotic effects on many types of chronic liver diseases via producing pro-fibrotic mediators, such as IL-4, IL-13 (61), osteopontin (OPN) (39), and Hh (62). Since the function of NKT cells in different stages and different etiologies of liver fibrogenesis displays the double-sword face, the net impact of NKT cells on liver fibrogenesis depends on the equilibrium between their anti-fibrotic effects and pro-fibrotic effects (63). Increasing evidence demonstrated that the function of NKT cells appears to be regulated by Hh signaling. Both mouse and human NKT cells express the ligand Shh, the receptor Ptc, and the transcription factor Gli1/Gli2, which indicates that NKT cells have the capability of producing and responding to Hh signaling (40). The recruitment and migration of peripheral NKT cells into injured liver tissues are controlled by CXCR6–CXCL16 chemokine axis (41), and the production and secretion of CXCL16 by immature cholangiocytes is dependent on Hh signaling (33). Moreover, *in vitro* study revealed that Hh ligands (Shh) can promote the proliferation and survival of NKT cells, which suggested that Hh signaling might exert anti-apoptotic effects on NKT cells (40). Therefore, the Hh (Shh)-rich microenvironment in chronic injured livers contributes to the recruitment of circulating NKT cells into the injured liver, prolongs their intrahepatic survival, and increases their opportunities for the exposure to potential antigen-presenting cells (e.g., activated HSCs). Meanwhile, the function of NKT cells on liver fibrogenesis is mainly mediated by Hh signaling. NKT cells are able to produce Hh ligands, which stimulate Q-HSCs to transdifferentiate into myofibroblasts responsible for the production of collagen matrix (62). Furthermore, pro-fibrogenic cytokines, such as IL-13 and IL-4, were secreted by Hh ligand-induced NKT cells (40). In particular, recent study demonstrated that the fibrosis progression in non-alcoholic steatohepatitis (NASH) is NKT cell dependent, and NKT cell-mediated fibrogenesis requires Hh and OPN, the latter of which, as an Hh-regulated cytokine, can further activate HSCs and promote fibrogenesis in an autocrine or paracrine fashion (39).

### Kupffer Cells/Macrophages and Hh

Liver macrophages display a remarkable heterogeneity and consist of resident Kupffer cells and infiltrating monocyte-derived macrophages. Kupffer cells are tissue-resident phagocytes with self-renewal capacity, which serve as critical surveillants that ensure liver homeostasis and eliminate antibodies, debris, or dead cells (64). Liver injury triggers Kupffer cells activation, and activated Kupffer cells release inflammatory cytokine and chemokine, which recruit peripheral blood monocytes into the injury liver (65). These infiltrating monocyte-derived macrophages rapidly expand the macrophage pool of the liver and further shape their phenotype depending on signals derived from the liver tissue microenvironment during various liver diseases (66, 67). In the fibrogenesis of chronic liver diseases, the injury-activated Hh signaling is also such a microenvironmental signal, which not only recruits peripheral monocytes into the liver but also modulates the phenotype and differentiation of liver macrophages. Kupffer cells are not only Hh-responsive cells but also Hh-producing cells, and injury-activated Kupffer cells can release Hh ligands, including Shh and Ihh (42). Moreover, peripheral blood monocytes, as the precursors of macrophages, also express components of Hh signaling (e.g., Ihh, Dhh, Ptc, Smo, SUFU, and Gli3), which suggest that monocytes can be susceptible to Hh signal stimulation (43). Furthermore, Shh, as a potent chemoattractant for monocytes, can activate classical signaling pathways associated with migration, such as PI3K or G-proteins (43). In chronic injured liver, activated Hh signaling not only recruits peripheral monocytes into the liver and amplify the intrahepatic macrophage pool but also modulates the phenotype of liver macrophages. During liver fibrosis progression in schistosomiasis, schistosome egg antigen (SEA) stimulates liver macrophages to produce and secrete Hh ligands with biological activity. Those Hh ligands act as chemoattractants for monocytes and also autocrinally induce the alternative activation of macrophages (42). Recent study in non-alcoholic fatty liver diseases (NAFLD) further showed that activated Hh signaling is able to regulate the production of OPN in hepatocytes through Gli1-dependent mechanism. These Hh-induced OPN can subsequently recruit bone marrow-derived monocytes into the liver and further paracrinally stimulate the pro-inflammatory phenotype of hepatic macrophages, which importantly contributes to the progression of NAFLD. Moreover, Hh inhibition (Smo antagonists or deletion of Smo in hepatocytes) has been shown to reduce hepatic inflammation and fibrogenesis induced by high-fat diet through inhibiting the activation of macrophages and the pro-inflammatory response mediated by macrophages (44).

### T Lymphocytes and Hh

Hepatic T lymphocytes show remarkable heterogeneity because of their diverse immunological functions and their different expression profiles of receptors. In general, there are two major kinds of T cells: CD4^+^ T cells (Th cells) and CD8^+^ T cells (cytotoxic T cells, CTLs), which perform multiple pro-inflammatory (or pro-fibrotic) or anti-inflammatory (or anti-fibrotic) functions in chronic liver diseases (68). Following liver injury, CD4^+^ Th cells, which can differentiate into Th1, Th2, Th17, or Treg cells based on the initial stimulus and liver microenvironment, play a vital role in regulating anti-inflammatory and pro-inflammatory responses, and accordingly can activate CD8^+^ T cell into fully functional CTLs, disseminate excessive inflammation, and then also impact liver fibrogenesis (68). *In vivo* and *in vitro* studies demonstrated that Hh signaling is of crucial importance for the development and differentiation of T cells in the thymus (7). Peripheral T cells also express components of Hh signaling pathway (48), and *in vitro* study demonstrated that Hh ligands (Shh) can modulate activation and cytokine production of peripheral CD4^+^ T cells (49). Moreover, recent study showed that upregulated Hh proteins derived from lung epithelial cells during allergic inflammation increased the production of IL-4 in local CD4^+^ Th cells, caused a shift toward a Th2-like profile, and then enhanced Th2 responses, which resulted in maintaining allergic inflammation and further aggravating disease (69). However, it is still unclear whether enriched Hh in the microenvironment of liver tissue injury and fibrotic repair are sufficient to modulate the differentiation and effector function of resident T cells as well.

### B Lymphocytes and Hh

It is initially considered that B lymphocytes do not participate in liver fibrogenesis. However, recent study in the carbon tetrachloride (CCl_4_)-induced fibrotic model of B cell-deficient mice showed that B cells are able to influence liver fibrogenesis in an antibody-independent and T cell-independent fashion (70). There are several possible mechanisms responsible for the pro-fibrotic effect of B cells on liver fibrosis: first, the pro-fibrotic cytokine IL-6 produced by B cells contributes to liver fibrogenesis through inducing the transdifferentiation of HSCs into myofibroblasts, promoting the proliferation of fibroblasts and enhancing the synthesis of collagen and tissue inhibitor of metalloproteinases (TIMPs); second, lack of autoantibody production in B cell-deficient mice may affect the activity of pro-fibrogenic cytokines and alter the function of T cells, which contributes to explaining why the absence of B cells can attenuate liver fibrosis (2). Although different studies demonstrated that Hh signaling pathway participates in the regulation of the development of B-lymphoid lineages (45), protecting germinal center B cells from apoptosis (46), and promoting B lymphopoiesis in a non-cell-autonomous fashion (47), it is still unclear whether enriched Hh existing in liver fibrotic tissues are sufficient to modulate local B cell effector function as well.

### Neutrophils and Hh

Neutrophils play a crucial role in eliminating invading organisms, but overwhelming activation of these cells may aggravate liver injury (71). Although there is lack of direct evidence for the regulation of Hh signaling on the function of neutrophils in liver fibrosis, existing evidence suggests that the recruitment of neutrophils into injured liver tissues appears to be closely related with Hh signaling. In a mice model of CCl_4_-induced liver fibrosis, the recruitment of neutrophils into the liver is mediated by CXCL2 in a S100A9-dependent manner (50). Hh signaling can stimulate cholangiocytes to express CXCL2 (33), and S100A9 is Gli target gene which has highly conserved Gli1-binding homologous sequences (51, 52). However, infiltrating neutrophils appear to play minor roles for liver fibrosis, because either the depletion of neutrophils or the dysfunction of neutrophils had not influenced the fibrogenic responses in mice following bile duct obstruction (72).

### Myeloid-Derived Suppressor Cells (MDSCs) and Hh

Myeloid-derived suppressor cells are identified as a heterogeneous population of immune cells that are characteristic with the capability for suppressing the activation of T cells through producing multiple immunosuppressive molecules (e.g., IL-10, TGF-β, and L-arginine) (73). MDSCs have been verified to exist in the normal liver (74), and under hepatic and extrahepatic diseases, the liver is also shown to a site of MDSC accumulation and differentiation (73). Growing data from experimental models and patients indicated that MDSCs have been involved in the pathogenesis of chronic liver diseases, including chronic hepatitis, fibrosis, and hepatocellular carcinoma (HCC) (73, 75). However, the exact role of MDSCs in chronic liver injury and fibrogenesis is still unclear. Recent study showed that MDSCs derived from bone marrow can produce IL-10 and impair the pro-fibrotic function of activated HSCs, and consequently alleviate liver fibrosis induced by CCl_4_ (76). On the contrary, the fibrogenic responses to chronic liver injury had not been affected in a CREM-α-overexpressing transgenic mouse model, which impairs the suppressive capacity of hepatic monocytic MDSCs (77). Although there is no direct evidence that Hh signaling can modulate the function of MDSCs in liver fibrosis, research on chronic infection caused by *Helicobacter pylori* and associated inflammation (53) suggested that the polarization of the invading myeloid cells to MDSCs requires the Hh-regulated transcription factor Gli1. Moreover, Gli1 mediated this phenotypic shift from the invading myeloid cells to gastric MDSCs by directly inducing a Gli1 target gene and myeloid differentiation factor Schlafen 4 (slfn4). Therefore, Hh signaling pathway participates in the polarization of the invading myeloid cells to MDSCs, which creates a microenvironment favoring wound healing and neoplastic transformation in gastric cancer. More studies need to be carried on in order to identify the precise role of Hh signaling in the function of MDSCs in liver fibrogenic repair.

### Hepatocytes and Hh

In addition to their essential metabolic roles, hepatocytes execute an abundance of important immunological functions. For example, hepatocytes can secrete plasma proteins (e.g., clotting factors, complement and antimicrobial proteins), generate acute phase proteins in the case of systemic or local infection, and present antigen to hepatic T cells (78). Moreover, hepatocyte death, triggered by various etiologies (e.g., virus infection, steatosis, or alcoholism), is generally viewed as the major cause of chronic liver inflammation and fibrosis. The apoptotic hepatocytes themselves have been shown to not only stimulate the production of pro-inflammatory and pro-fibrogenic cytokines by macrophages but also directly activate HSCs (79–81). Meanwhile, it has been reported that multiple damage-associated molecular patterns (DAMPs) (82) and inflammatory cytokines (e.g., IL-33) (83) released from dying hepatocytes also may promote hepatic inflammation and fibrogenesis. Recent data further indicated that pro-apoptotic stimuli provoke mature hepatocytes to produce Hh ligands (e.g., Shh and Ihh) (23). These Hh ligands act on neighboring cells in a paracrine manner, and many neighboring cells that are mostly engaged in liver remodeling, including myofibroblasts, liver progenitors, HSCs, immature cholangiocytes, LSECs, and immune cells, are Hh-responsive cells (6). These Hh-responsive cells further amplify Hh signaling cascade, and meanwhile promote hepatic inflammation and fibrogenesis.

### Cholangiocytes and Hh

Cholangiocytes were initially considered to be simple columnar epithelia which constitute the intrahepatic and extrahepatic bile ducts (84). Accumulating experimental data unveiled that cholangiocytes are dynamic and critical to both innate and adaptive immune responses of the liver, which mainly reflects on defensing against foreign substances and releasing inflammatory modulators in a regulated manner (84). Especially, release of inflammatory cytokines and chemokines by cholangiocytes, which mediates the infiltration of inflammatory cells in the ductular reaction, is vital for the fibrogenesis of chronic cholestatic liver disease. Cholangiocytes are not only Hh-producing cells but also Hh-responsive cells (25). During chronic biliary injury, the injury-related activation of Hh signaling has been displayed for stimulating cholangiocytes to secrete several chemokines, including for neutrophils (CCL20/MIP3α, CXCL1, CXCL2, and CXCL5), for monocytes/macrophages (CCL2/MCP-1 and CCL20/MIP3α), and for lymphocytes (CCL20/MIP3α and CXCL11) (33). These chemokines are able to recruit, attract, and retain these aforementioned immune cells into injured liver tissues, which can initiate and amplify the inflammatory response (33). Furthermore, these infiltrating inflammatory cells, in turn, produce more Hh ligands and amplify the Hh signaling cascade. Meanwhile, these cells also produce pro-fibrogenic cytokines, such as IL-6, IL-4, IL-13, and TGF-β1, which mediate duct injury and repair, and myofibroblastic transdifferentiation of portal fibroblasts (84).

### LSECs and Hh

Liver sinusoidal endothelial cells are regarded as highly specialized endothelial cells displaying distinctive morphological phenotype and function, and constitute the walls of liver sinusoids (85). In patients and animal models, capillarization of LSECs, which is characterized as the disappearance of fenestration associated with basement membrane formation and change in surface marker expression, occurs following liver injury (85). During liver fibrogenesis, the capillarization of LSECs precedes the activation of HSCs and macrophages and therefore is viewed as an initial pathologic change that could be necessary for fibrogenic repair (86, 87). Capillarized LSECs lose their abilities to maintain the quiescence of HSCs, which could promote intrahepatic vasoconstriction and the development of fibrosis (88). LSECs are also both Hh-producing cells and Hh-responsive cells (30), and Hh signaling has been verified to regulate LSEC capillarization. During the process of capillarization, Hh signaling in LSECs is activated in accompany with downregulated expression of Hhip and upregulated expression of Hh-regulated genes (55). *In vitro* and *in vivo* study further demonstrated that inhibiting Hh signaling not only could completely prevent capillarization but also could partially revert dedifferentiated LSECs to their differentiated phenotype (55). These data suggested that LSEC capillarization is an Hh-dependent process. Moreover, *in vitro* study demonstrated that Hh signaling is required for the migration of LSECs and vascular tube formation, because Hh pathway inhibitors can block both LSECs migration and tube formation induced by VEGF whereas Hh pathway agonists can enhance these processes. This is partly explained that Hh signaling can regulate VEGFR1 and VEGFR2 expression on LSECs (55).

In addition, during liver fibrogenic repair, HSCs have been initially identified as the major effector cells because of their ability to transdifferentiate into collagen-producing myofibroblasts (32). More recently, evidence elucidates that HSCs, particularly activated HSCs, also have a fundamental role for the local immunity in the liver. Activated HSCs mediate a range of immunoregulatory effects by producing various inflammatory-associated mediators (e.g., NADPH oxidase enzymes and reactive oxygen species, pro-inflammatory cytokines, and chemokines); by expressing chemokine receptors (including CCR5, CCR7, CXCR3, and CXCR7); by responding to bacterial components through TLR4; and by serving as non-professional antigen-presenting cells in injured liver (89). Moreover, these immunoregulatory effects mediated by activated HSCs, as well as the direct or indirect interactions between HSCs and different immune cell subtypes, function together to promote liver fibrogenesis. Q-HSCs are Hh-responsive cells, whereas activated HSCs are Hh-producing cells (30). In liver fibrogenesis, the injury-activated Hh signaling can activate Q-HSCs, transdifferentiate Q-HSCs into MF-HSCs, promote the proliferation of MF-HSCs, and prolong the survival of MF-HSCs (17, 22). That is, Hh signaling is capable of inducing and retaining immune phenotype of HSCs. Moreover, activated HSCs-derived Hh ligands, in turn, amplify the Hh signaling cascade in liver fibrotic tissues and further promote the immune and pro-fibrotic phenotype transition of Q-HSCs. Meanwhile, as mentioned above, Hh signaling also mediates the cross-talking between HSCs and other immune cells, such as the cross-talking between HSCs and NKT cells, in liver fibrotic tissues (40, 62).

## Targeting Hh Signaling Pathway for Anti-Fibrotic Therapy through Modulating Liver Inflammation

Recently published literature describes Hh pathway inhibitors as the following three categories: inhibitors targeting Smo, including nature compound (cyclopamine) and synthetic small molecules (vismodegib, sonidegib, IPI-926, BMS-833923, PF-04449913, SANT1–4, Cur61414, MRT-10); strategies to target the upstream N-Shh ligand, including Robotniknin and anti-Shh 5E1; and inhibitors targeting the downstream transcription factors Gli1 and Gli2, including Darinaparsin, GANT-61/58, HPI-1, and Forskolin (90). Various *in vivo* and *in vitro* studies demonstrated that some of these Hh pathway inhibitors, such as vismodegib (91), sonidegib (92), Forskolin (93), GANT-61 (94), GANT-58 (95), and cyclopamine (96), are capable of inhibiting or reversing the progression of liver fibrosis. Moreover, modulating repair-related inflammation response during the liver injury and repair is one of the important mechanisms of action for anti-fibrotic effect of these Hh pathway inhibitors. Study based on various rodent models of NASH induced by diet suggested that specific Smo inhibitors (vismodegib or sonidegib) suppressed the activity of Hh signaling and accordingly alleviated the degree of fibrosis, mainly by attenuating macrophage-associated and NKT-related liver inflammation (39, 44, 97, 98). Consistent with the above data in NASH, the anti-fibrotic effect of vismodegib was evidenced by significant amelioration of hepatic schistosomal fibrosis in a murine experimental model, which was associated with the significant reduction in granuloma diameter and amelioration of the liver tissue affection seen in mild inflammatory cellular infiltrate and portal tract affection (91). Moreover, *in vitro* studies demonstrated that two different pharmacologic antagonists of Smo (cyclopamine or vismodegib) also alleviated SEA-induced liver inflammation response by inhibiting the alternative activation of macrophages and consequently contributed to limiting the progression of schistosomiasis fibrosis (42). In addition, in a rat model of CCl_4_-induced liver fibrosis, another Hh pathway inhibitor Forskolin can inhibit the Hh signaling pathway through cAMP-mediated activation of PKA, the latter of which, in turn, caused further attenuation of inflammation, repression of HSC activation, and, consequently, decreased liver fibrogenesis (93).

More importantly, Hh signaling has been involved in both liver fibrosis and HCC. It has been proposed that the excessive Hh signaling of HSCs and inflammatory cells, as well as progenitors in injured liver, contributes to the occurrence and development of HCC (23, 99). Recent study based on a Shh-transgenic mouse model further revealed that the aberrant activation of Hh signaling in the stromal cells and immune cells contributes to creating a microenvironment favoring the malignant transformation of hepatocytes during liver fibrogenesis (100). This seems to, to some extent, explain why chronic liver injury in accompany with fibrogenesis is a main risk factor for the occurrence of HCC. Moreover, treatment with vismodegib in the Mdr2^−^/^−^ mouse model of progressive liver fibrosis and spontaneous hepatocarcinogenesis significantly relieved the degree of fibrosis, induced the involution of intrahepatic HCC, and reduced the number of metastatic HCC through inhibiting hepatic Hh signaling activity and decreasing liver myofibroblasts and progenitors without increasing mortality (101). Therefore, Hh-targeted pharmacological interventions might serve as a potential therapeutic strategy not only for liver fibrosis but also for fibrosis-associated HCC.

## Conclusion and Future Directions

Since anti-fibrotic strategies based on manipulating the immune system are beginning to emerge in the clinical field (38), it is timely and necessary to clarify how immune responses may be subtly modified in liver tissue remodeling. The conception that Hh, as a morphogen existing on liver injury microenvironment, could manipulate the immunologic aspects of liver fibrotic repair and affect the pathologic inflammatory milieu within liver fibrogenic tissues, is now more and more strongly supported by fantastic amounts of data. This largely contributes to enlarging our understanding about the pathogenesis of liver fibrosis and promotes the development of Hh-centered therapeutic approaches. Although Hh signaling pathway is shown to hold central roles for promotion of liver inflammation and fibrosis, as well as in the transition from chronic injury to hepatocarcinogenesis, the precisely molecular determinants in mediating these roles are to a large extent unknown. Meanwhile, more details about how Hh signaling is modulated by and interacts with other morphogen signaling (e.g., Wnt and Notch) that also regulate adult liver repair should be further investigated. In addition, more studies should be required in order to evaluate the efficacy of Hh pathway inhibitors as anti-fibrotic agents in various experimental liver fibrosis. If successful, it might pave the way to establishing Hh-centered therapies for liver fibrosis and fibrosis-associated HCC, especially both the effectiveness of these inhibitors in solid organ tumors (e.g., advanced basal cell carcinoma) (102) and their human safety profile (103, 104) have been verified in clinical trials. Taken together, based on the aforementioned data, we conclude that detailed knowledge of modulating the repair-associated inflammation by the injury-related activation of Hh signaling pathway might deepen our understanding of the relationship between inflammation and fibrosis underlying liver injury and repair and have potential clinical application in the therapy of liver fibrosis.

## Author Contributions

XS and HL researched the topic. XS and YP wrote and edited the manuscript. All authors have read and approved the final manuscript.

## Conflict of Interest Statement

The authors declare that the research was conducted in the absence of any commercial or financial relationships that could be construed as a potential conflict of interest.
